# Investigating the potential role of swertiamarin on insulin resistant and non-insulin resistant granulosa cells of poly cystic ovarian syndrome patients

**DOI:** 10.1186/s13048-023-01126-0

**Published:** 2023-03-18

**Authors:** Muskaan A. Belani, Preeti Shah, Manish Banker, Sarita S. Gupta

**Affiliations:** 1 Dr. Vikram Sarabhai Institute of Cell and Molecular Biology, Faculty of Science, The Maharaja Sayajirao University of Baroda, Vadodara, Gujarat 390 002 India; 2 Nova IVI Fertility, Behind Xavier’s Ladies Hostel, 108, Swastik Society Rd, Navrangpura, Ahmedabad, 390009 Gujarat India

## Abstract

**Background and aim:**

Conventional drugs have limitations due to prevalence of contraindications in PCOS patients. To explore the potential effects of swertiamarin, on abrupted insulin and steroidogenic signaling in human luteinized granulosa cells from PCOS patients with or without insulin resistance.

**Experimental procedure:**

hLGCs from 8 controls and 16 PCOS patients were classified for insulin resistance based on down regulation of protein expression of insulin receptor-β (INSR- β) as shown in our previous paper. Cells were grouped as control, PCOS-IR and PCOS-NIR, treated with swertiamarin (66 µM) and metformin (1 mM). Expression of key molecules involved in insulin signaling, fat metabolism, IGF system and steroidogenesis were compared between groups.

**Results:**

Swertiamarin significantly (*P* < 0.05) reversed the expression of INSR-β, PI(3)K, p-Akt, PKC-ζ, PPARγ, (*P* < 0.01) IRS (Ser 307) and IGF system in PCOS-IR group and was equally potent to metformin. In the same group, candidate genes viz SREBP1c, FAS, ACC-1 and CPT-1 were down regulated by swertiamarin (*P* < 0.001) and metformin (*P* < 0.001). Significant upregulation was demonstrated in expression of StAR, CYP19A1, 17β-HSD and 3β-HSD when treated with swertiamarin (*P* < 0.01) and metformin (*P* < 0.01) in PCOS-IR followed by increase in 17β-HSD and 3β-HSD enzyme activity along with estradiol and progesterone secretions. However, swertiamarin did not reveal any effect on PCOS-NIR group as compared to metformin that significantly (*P* < 0.01) reversed all the parameters related to steroidogenesis and down regulated basal expression of insulin signaling genes.

**Conclusion:**

Swertiamarin, presents itself as a potential fertility drug in hLGCs from PCOS-IR patients.

**Supplementary Information:**

The online version contains supplementary material available at 10.1186/s13048-023-01126-0.

## Introduction

Polycystic ovarian syndrome (PCOS) is a multifaceted disease and an approach to decrease this has become a top priority for many health organizations [1]. The life style factors such as physical exercise, psychological stress, high carbohydrate diet and sedentary life that impact fertility are modifiable and are considered to be the first-line of treatment in PCOS [2–4]. Symptom oriented pharmacological intervention such as oral contraceptive to regulate menstrual cycles and decrease hirsutism, spironolactone that block androgen receptors, finasteride to block production of active form of testosterone and insulin sensitizers such as metformin, thiazilidinediones (TZD’s) and D-chiro Inositol are in use for improving insulin sensitivity and lipid levels accompanied by weight loss [1, 5, 6]. Psychosocial interventions have also proven to improve PCOS-related mental health issues [7].

Considering the role of insulin resistance (IR) in the interplay of metabolic and reproductive aberrations in infertility, insulin sensitizing drugs (ISD) are expected to have beneficial effects by restoring ovulatory menstrual cycles, reducing insulin resistance and thus being important therapeutic modality for PCOS [8, 9]. Insulin sensitizers such as the thiazolidinediones (TZD’s), D-chiro-Inositol, and metformin are postulated to improve insulin sensitivity and several other aspects of the syndrome, including reproductive abnormalities [10, 11]. TZD’s improve insulin sensitivity in PCOS through decrease in hepatic glucose production [11]. Studies with TZD’s have reported fetal growth restriction as a potential risk in animal experiments and high incidence of weight gain among the users that further hampers their use in obese women with PCOS [12, 13]. D-chiro-Inositol has been observed to show effect on serum hormone binding globulin levels without affecting testosterone, fasting glucose, fasting insulin, and ovulation rate [14]. Supplementation of insulin sensitizer myo-inositol has demonstrated positive effects in oocyte quality of PCOS patients and post-menopausal transitions [15–17].

However, it’s role in improving clinical pregnancy rates is under controversy and is not recommended for normo insulinemic lean or obese PCOS [11, 18, 19]. Thus, none of the ISD’s could increase a chance of having a live birth and are limited by the prevalence of contraindications in women with PCOS [1, 10, 20]. Moreover with limited understanding of their mechanism, the type of patients, whether only PCOS-IR or even PCOS-NIR would be benefitted by their effects remains to be identified [21].

A number of herbal medicines such as Vitex agnus-castus, Cimicifuga racemosa, Tribulus terrestris, Glycyrrhiza spp*., Paeonia lactiflora, Cinnamomum cassia and *Aloe vera* can improve ovarian function, androgen excess, obesity, insulin resistance, blood lipids and inflammation and exert beneficial effects in PCOS [1, 6, 22–24]. *Enicostemma Littorale blume* is a herbal plant that is known for its anti-diabetic effect but its bio-active molecules has been unexplored in the field of PCOS.

*Enicostemma Littorale blume* (EL) has been widely used by folks since ages for the treatment of diabetes. During last ten years our lab worked on EL and unravelled its hypo gycemic, hypo lipidemic, anti-inflammatory and insulin sensitizing potentials in different animal models [25–28]. Swertiamarin, being its principal compound has proved to be a potent insulin sensitizer in STZ-NA diabetic rat models and different cell lines [29, 30]. The present study focuses to investigate direct effect of bio-active molecule swertiamarin on reproductive endocrinology for the treatment of women with PCOS.

Certain obese and lean women with PCOS are observed to have normal insulin sensitivity, hence it would be interesting to discover potential bio actives for treating PCOS-IR and NIR, considering the side effects observed by conventional pharmaceutical drugs. [31]. PCOS patients were classified as IR and NIR based on a novel molecular marker insulin receptor – β (INSR-β) expression on luteinized granulosa cell as shown in our previous paper [32]. The effects of swertiamarin and metformin have been explored on granulosa cell death, insulin and steroidogenic signaling, genes involved in fatty acid metabolism and steroidogenic hormones in granulosa cells from PCOS-IR and PCOS-NIR.

## Materials and methods

The study was approved by the Institutional Ethics committee for human research (IECHR), Faculty of Science, The M. S. University of Baroda, Vadodara (Ethical Approval Number FS/IECHR/BC/SG2).

### Human follicular fluid

Human follicular fluid samples were collected after informed consent from patients undergoing IVF/ ICSI over the course of 12 months at Nova IVI Fertility Clinic, Ahmedabad, India from 2015 August TO 2016 April. All the controls and patients underwent controlled ovarian hyper stimulation (COH) using flexible antagonist protocol. Follicular fluid was sent in embryology laboratory for oocyte identification & oocytes were separated out for IVF/ICSI. The follicular fluid devoid of oocyte was collected for the experiments. All the controls and patients received a GnRH analog (GnRH-a) in combination with FSH or human menopausal gonadotropin (hMG), followed by administration of human chorionic gonadotropin (hCG). The follicular fluid was collected on the day of oocyte retrieval.

#### Inclusion criteria

The diagnosis included donors, male factor infertility, tubal factor infertility and PCOS with an age ranging from 20–40 years.

#### Exclusion criteria

Patients with endometriosis and poor ovarian response were excluded from the study.

### Human granulosa-luteal cell culture with bioactives

Luteinized granulosa cells from individual control and PCOS follicular fluid aspirates were isolated, analyzed for expression of INSR-β and segregated as control, PCOS-IR and PCOS-NIR as done before [32]. The cells from *n* = 8 control, *n* = 8 PCOS-IR and *n* = 8 PCOS-NIR were pooled in respective groups and cultured in at a density of 0.5 × 10^6^ cells in 3 ml of DMEM/F12 culture medium supplemented with 10% FBS and Penicillin-G/ Streptomycin (100 IU/ml/100 mg/ml) in a 6-well plate at 37° C and 5% CO_2_ in a humidified incubator for 48 h. The cells were then incubated in serum free culture medium with or without swertiamarin (66 μM) and positive control- metformin (1 mM) for additional 72 h. The doses were selected based upon the studies in literature [30, 33, 34]. The viability of cells was analysed after 72 h by trypan blue exclusion dye method following which the supernatant was collected for hormone analysis and cells harvested for gene and protein expression and enzyme activity.

### Total RNA Extraction, RT-PCR and qRT-PCR

Total cellular RNA was isolated from the cultured luteinized granulosa cells and then reverse transcribed into first strand cDNA. qRT-PCR for lipogenic genes Sterol Regulatory Element Binding Protein (SREBP1c), Fatty acid synthase (FAS), Acetyl-CoA carboxylase 1 (ACC-1), Carnitine palmitoyl transferase I (CPT-1), Insulin like growth factor system (IGF-1, IGF-2, IGF-1R, IGF-2R) and gonadotropins receptors [follicle stimulating hormone receptor (FSH-R), luteinizing hormone receptor (LH-R)] study was performed on an Applied- Biosystem 7500-Real-Time PCR Sequence detection System using standard temperature cycling conditions. qRT-PCR for steroidogenic genes Steroidogenic Acute Regulatory protein (StAR), Cytochrome P450 side chain cleavage (CYP11A1), 3-beta hydroxy steroid dehydrogenase (3 β- HSD), Aromatase (CYP19A1), 17-beta hydroxy steroid dehydrogenase (17 β-HSD) was performed on Applied Biosystem 7500 FAST Real Time PCR. Sequence detection System by predesigned primers from TaqMan gene expression assays. All qRT-PCR results were normalized to the level of β-actin and 18S rRNA determined in parallel reaction mixtures to correct any differences in RNA input. Fold changes in qRT-PCR gene expression were analyzed using 7500 Real time PCR software V.2.0.6 and Data assist software (Applied Biosystems Inc.) which led to a possible estimation of the actual fold change. Negative RT was performed with untranscribed RNA. (Supplementary files for Primer sequence).

### Western blot analysis

The cultured luteinized granulosa cells were harvested suspended in cell lysis buffer composed of 62.5 mM Tris–HCl, pH 6.8, 6 M urea, 10% (v/v) glycerol, 2% (w/v) SDS, 0.00125% (w/v) bromophenol blue and freshly added 5% (v/v) β-mercaptoethanol and subjected to sonication on ice. Total protein content was quantified using Bradford assay (Biorad Bradford Solution, USA). 20 µg protein was loaded on 10% SDS–polyacrylamide gel electrophoresis under reducing conditions, along with pre-stained molecular weight markers. The separated proteins were electrophoretically transferred onto a nitrocellulose membrane and incubated overnight at 4 °C with appropriate antibody dilution (supplementary file for table). The samples were then incubated for 1 h at room temperature with a horseradish peroxidase-conjugated anti-rabbit or anti-goat or anti-mouse IgG and analyzed by Alliance 4.7 UVI Tec chemidoc.

### Hydroxysteroid dehydrogenase activity

17β-HSD and 3β-HSD activities were estimated in cultured granulosa cells following Shivanandappa &Venkatesh [35]. In brief, the assay system contained 0.1 M Tris–HCl (pH 7.8), 5 mM nicotinamide adenine dinucleotide (NAD), 1 mM estradiol/dehydroepiandrostenedione (DHEA), and 0.4 mM 2-p-iodophenyl-3-p-nitrophenyl-5-phenyl tetrazolium chloride (INT) and 50 µl of granulosa cell lysate containing enzyme in a total volume of 3 ml, which was incubated for 1 h at 37 °C. The reaction was terminated using 50 mM potassium phthalate buffer, and absorbance was measured at 490 nm.

### Hormone analysis

The steroid hormones were measured from the culture medium by enzyme-linked immunosorbent assay (Diametra; Italy), according to the manufacturer’s instructions. The standard curve for E2, P4 and T ranged from 0 to 2000 pg/mL, 0 to 40 ng/ml and 0 to 16 ng/ml respectively. The supernatants were diluted to 1: 1000 for E2 and 1: 250 for P4 in PBS to ensure that the final value fell within the detection range of the standard curve. Each sample was assayed in duplicate, and the E2 and P4 concentration was calculated by multiplying the end value by the dilution factor. The assay sensitivity range was 8.68 pg/ml for E2, 0.05 ng/ml for P4 and 0.07 ng/ml for testosterone.

### Statistical analysis

The results are presented as mean ± standard error mean. The data were statistically analyzed by employing one-way analysis of variance followed by Newman Keuls Multiple Comparison Test (GraphPad Prism; Graph Pad Software, Inc., La Jolla, CA). The minimum level of significance (*P* < 0*.*05) was considered. (1637 words).

## Results

### Swertiamarin increases hLGC viability from PCOS-IR only.

**Fig. 1:**
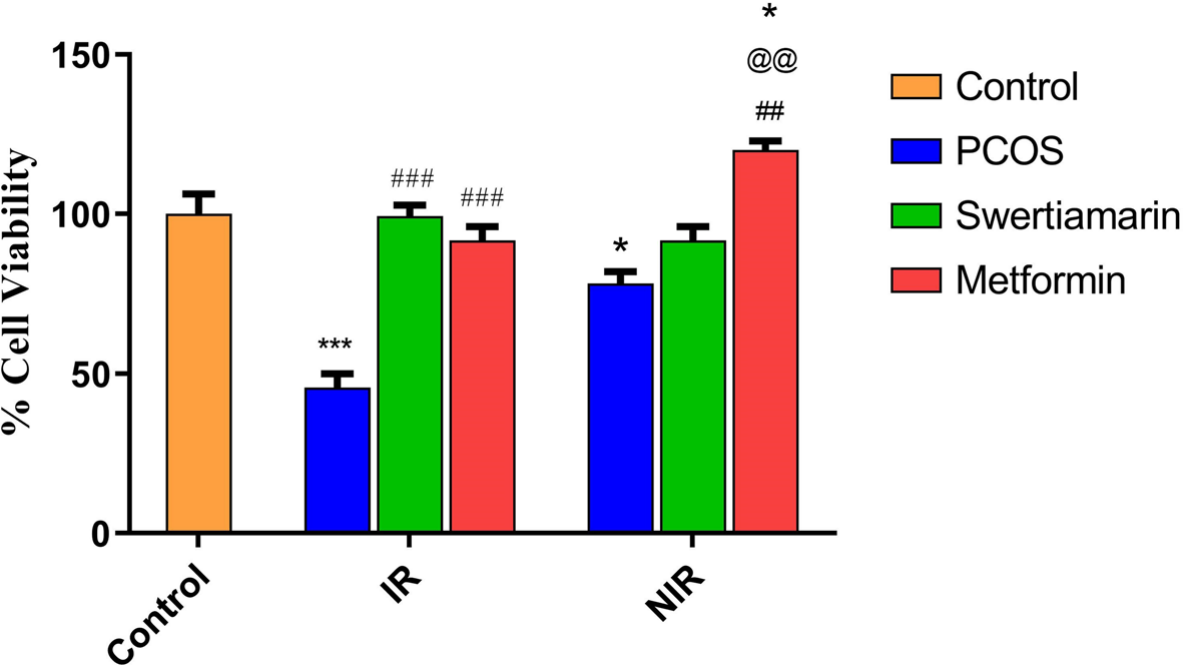
Effect of swertiamarin and metformin on cell viability in PCOS-IR and PCOS-NIR. % granulosa cell viability was done by trypan blue exclusion dye method. The normalized expression values are represented as mean ± SEM of three independent experiments. * *P* < 0.05, ** *P* < 0.01 vs. C, ^##^ *P* < 0.01 vs. PCOS-NIR, ^###^ *P* < 0.001 vs. PCOS-IR, ^@@^ *P* < 0.01 vs. PCOS-NIR swertiamarin *n* = 8 control, *n* = 8 PCOS-IR and *n* = 8 PCOS-NIR

Treatment of swertiamarin significantly (*P* < 0.001) ameliorated cell death in PCOS-IR group but no difference was observed because of these treatments in PCOS-NIR group. However, metformin markedly increased the viability in PCOS IR as well as PCOS-NIR group (Fig. 1).

### Divergent effects of swertiamarin on insulin signalling and  lipid metabolism 

**Fig. 2:**
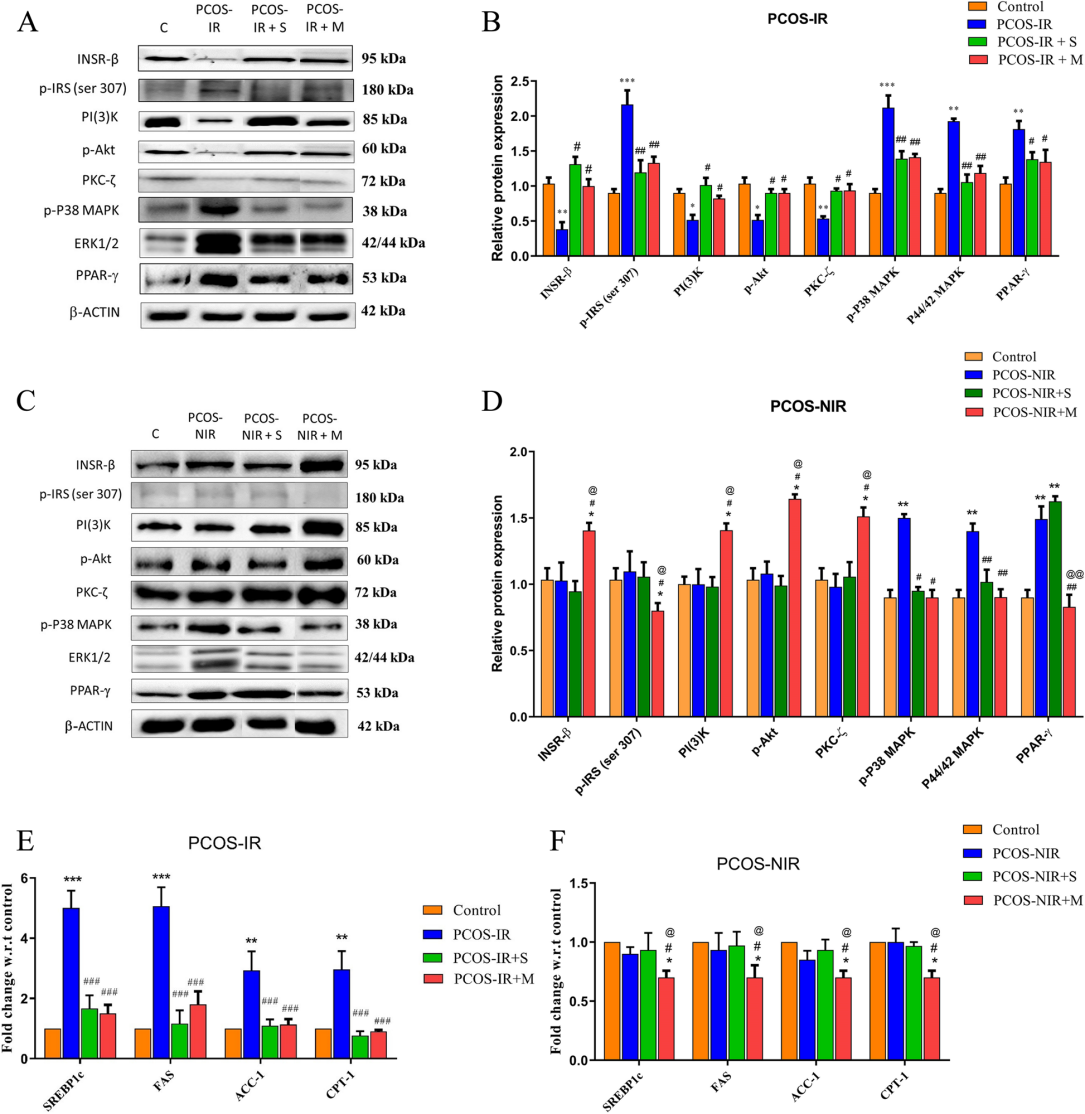
Expression of genes and proteins involved in insulin signaling cascade in human luteinized granulosa cells isolated from control, PCOS-IR, PCOS-NIR and treated with swertiamarin and metformin. **A** Western blot image for INSR-β, pIRS(ser307), PI(3)K, pAkt, PKC-ζ, ERK1/2, pP38MAPK and PPAR γ from PCOS-IR. **B** Densitometric analysis for INSR-β, pIRS (ser307), PI(3)K, pAkt, PKC-ζ, ERK1/2, pP38MAPK and PPAR γ from PCOS-IR. **C** Western blot image for INSR-β, pIRS(ser307), PI(3)K, pAkt, PKC-ζ, ERK1/2, pP38MAPK and PPAR γ from PCOS-NIR. **D** Densitometric analysis for INSR-β, pIRS (ser307), PI(3)K, pAkt, PKC-ζ, ERK1/2, pP38MAPK and PPAR γ from PCOS-NIR. **E** mRNA expression of SREBP1c, FAS, ACC-1, CPT-1 genes involved in fatty acid metabolism by qRT-PCR PCOS-IR. F. mRNA expression of SREBP1c, FAS, ACC-1, CPT-1 genes involved in fatty acid metabolism by qRT-PCR PCOS-NIR. * *P* < 0.05, ** *P* < 0.01 vs. C, ^#^ *P* < 0.05, ^##^ *P* < 0.01, ^###^ *P* < 0.001 vs. PCOS-IR or PCOS-NIR, ^@^ *P* < 0.05 vs. PCOS-IR + S, *n* = 8 control, *n* = 8 PCOS-IR and *n* = 8 PCOS-NIR

The possible effect of swertiamarin on protein expression of candidate insulin signalling intermediates viz insulin receptor (INSR-β), phospho insulin receptor substrate [pIRS(ser307)], phosphatidylinositol 3-kinase (PI(3)K), phospho protein kinase B (pAkt), protein kinase C (PKC-ζ), extracellular regulatory kinase (ERK1/2), phospho P38 mitogen activated protein kinase (pP38MAPK) and Peroxisome proliferator-activated receptor gamma (PPAR-γ) in PCOS-IR and NIR hLGCs were analyzed by western blot. Swertiamarin treatment significantly (*P* < 0.05) reversed the down regulated expression of INSR-β, PI(3)K, p-Akt and PKC-ζ in PCOS-IR hLGCs. Increased expression of p-IRS(Ser 307) (*P* < 0.01) a hall mark of insulin resistance and PPARγ (*P* < 0.05) – an indicative of PCOS was also reduced remarkably because of the treatment with these bioactive compounds in PCOS-IR cells. Swertiamarin could significantly decrease protein expression of pP38 MAPK and p44/42 MAPK in hLGC’s from both PCOS-IR as well as PCOS-NIR. Moreover, swertiamarin with 66 μM was observed to be equally potent to metformin 1 mM in PCOS-IR, but, surprisingly, swertiamarin was unable to show any effect on the candidate insulin signalling from PCOS-NIR (Fig. 2, A, B, C and D). Swertiamarin (*P* < 0.001) significantly down regulated the candidate genes viz SREBP1c, FAS, ACC-1 and CPT-1 in PCOS-IR as compared to PCOS-NIR where it did not show any effect. Here, swertiamarin was observed to be equally potent to metformin. Metformin down regulated the basal expression of all the genes in PCOS- NIR hLGC’s (Fig. 2, E and F).

### Divergent effects of swertiamarin on IGF system

**Fig. 3:**
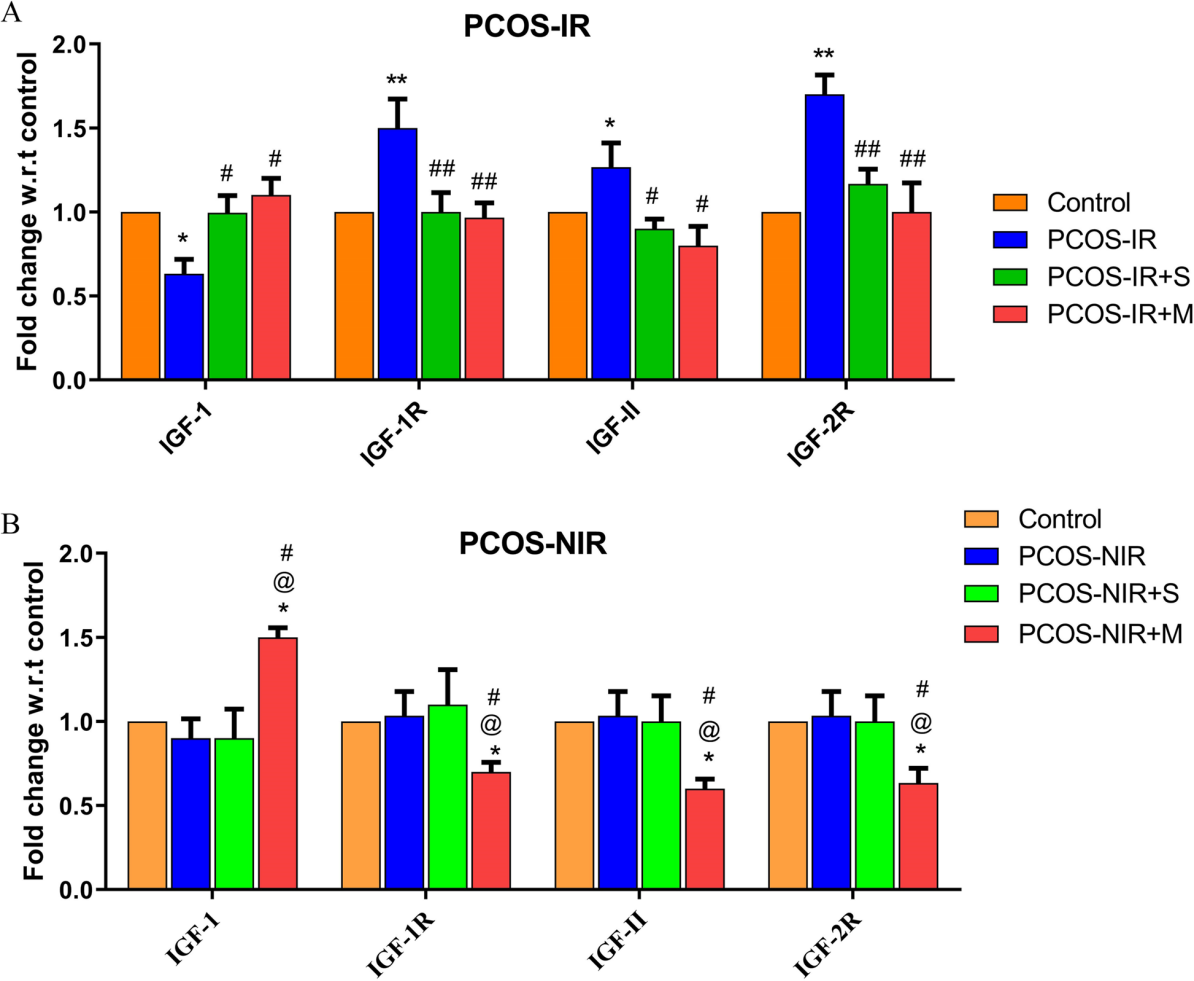
Expression of genes and proteins involved in IGF system in human luteinized granulosa cells isolated from control, PCOS-IR, PCOS-NIR and treated with swertiamarin and metformin. **A** mRNA expression of genes involved in IGF system in PCOS-IR. **B** mRNA expression of genes involved in IGF system in PCOS-NIR * *P* < 0.05, ** *P* < 0.01 vs. **C**, ^#^ *P* < 0.05, ^##^ *P* < 0.01, ^###^ *P* < 0.001 vs. PCOS-IR or PCOS-NIR, ^@^ *P* < 0.05 vs. PCOS-IR + S, *n* = 8 control, *n* = 8 PCOS-IR and *n* = 8 PCOS-NIR

Swertiamarin treatment could significantly (*P* < 0.05) upregulate the gene expression of IGF-1 in PCOS-IR group. The bioactive molecule appreciably down regulated the expression of IGF-1R (*P* < 0.01), IGF-II (*P* < 0.05) and IGF-2R (*P* < 0.01) in hLGC’s from PCOS-IR. Although there was no alteration in the IGF system in PCOS NIR group by swertiamarin; metformin treatment dramatically reduced the basal levels of all the genes in this group (Fig. 3, A and B).

### Swertiamarin re-establishes steroidogenesis in hLGC’s from PCOS-IR only

**Fig. 4:**
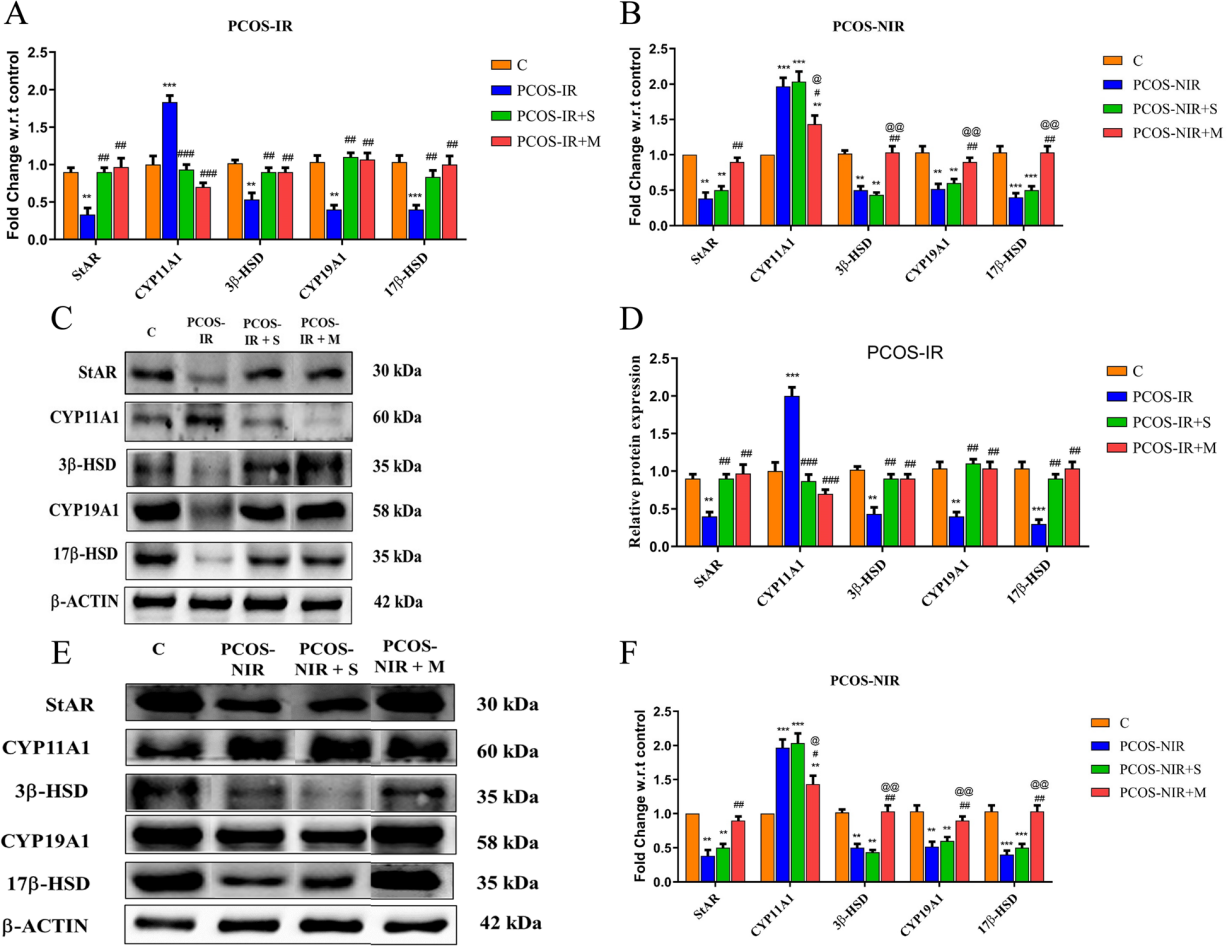
Steroidogenic profile of human luteinized granulosa cells isolated from control, PCOS-IR and PCOS-NIR and treated with swertiamarin and metformin. **A** mRNA expression *of* StAR, CYP11A1, 3β-HSD, CYP19A1, 17β-HSD genes in PCOS-IR by qRT-FAST PCR **B** mRNA expression *of* StAR, CYP11A1, 3β-HSD, CYP19A1, 17β-HSD genes in PCOS-NIR by qRT-FAST PCR. **C** Protein expression of StAR, CYP11A1, 3β-HSD, CYP19A1, 17β-HSD by Western blot method from PCOS-IR. **D** Densitometry analysis using β-actin as endogenous control from PCOS = IR. **E** Protein expression of StAR, CYP11A1, 3β-HSD, CYP19A1, 17β-HSD by Western blot method from PCOS-NIR. **F** Densitometry analysis using β-actin as endogenous control from PCOS-NIR. * *P* < 0.05, ** *P* < 0.01, *** *P* < 0.001 vs. C, ^#^ *P* < 0.05, ^##^ *P* < 0.01, ^###^ *P* < 0.001 vs. PCOS-IR or PCOS-NIR, ^@^ *P* < 0.05, ^@@^ *P* < 0.01, ^@@@^ *P* < 0.001 vs. PCOS-IR + S. *n* = 8 control and *n* = 8 PCOS-IR

Swertiamarin (*P* < 0.01) significantly up regulated mRNA as well as protein expression of StAR, CYP19A1, 17β-HSD and 3β-HSD and was equally potent to metformin in PCOS-IR. CYP11A1 demonstrated a significant down regulation in mRNA as well as protein expression in both the groups. Swertiamarin did not show any effect in PCOS-NIR condition. However, the golden drug metformin did show significant reversal (*P* < 0.01) of all the genes and proteins in PCOS-NIR group (Fig. 4, A, B, C, D, E and F).

### Swertiamarin reverts back enzyme activity and hormonal levels in hLGC’s from PCOS-IR only

**Fig. 5:**
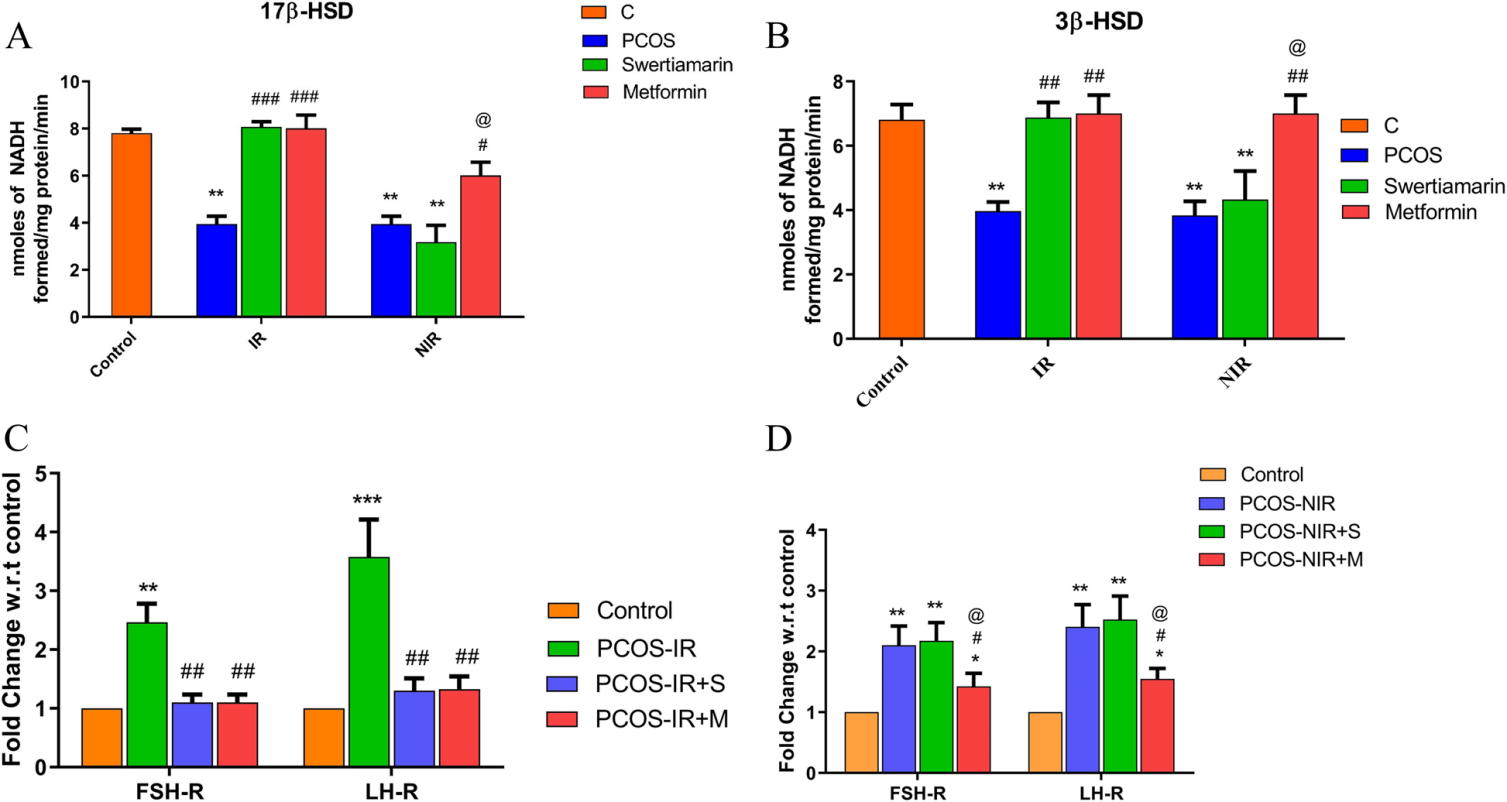
**A** Enzyme activity analysis of 3β-HSD and 17β-HSD in PCOS-IR and PCOS-NIR. **B** Analysis of steroid hormones estradiol, progesterone and testosterone concentration in follicular fluid aspirates devoid of luteinized granulosa cells by ELISA from PCOS-IR and PCOS-NIR. **C** and **D**) mRNA expression of FSH-R and LH-R genes in human luteinized granulosa cells by qRT-PCR from PCOS-IR and PCOS-NIR. * *P* < 0.05, ** *P* < 0.01, *** *P* < 0.001 vs. C, ^#^ *P* < 0.05, ^##^ *P* < 0.01, ^###^ *P* < 0.001 vs. PCOS-IR or PCOS-NIR, ^@^ *P* < 0.05, ^@@^ *P* < 0.01, ^@@@^ *P* < 0.001 vs. PCOS-IR + S. *n* = 8 control and *n* = 8 PCOS-IR

The activities of the enzymes 17β-HSD and 3β-HSD were also up regulated by swertiamarin (*P* < 0.01). A significant increase in 3β-HSD and 17β-HSD was demonstrated by treatment with swertiamarin (*P* < 0.01, *P* < 0.001) and metformin (*P* < 0.01, *P* < 0.001) in PCOS- IR hLGCs with respect to control, whereas in PCOS-NIR group only metformin demonstrated a significant increase (*P* < 0.01) in 3β-HSD activity as well as 17β-HSD activity (Fig. 5, A and B). In vitro secretion of estradiol, progesterone and testosterone were analysed from the conditioned media of PCOS-IR and PCOS-NIR treated with swertiamarin and metformin. A significant increase was demonstrated by treatment with swertiamarin (*P* < 0.001) and metformin (*P* < 0.001) in PCOS-IR hLGCs with respect to untreated PCOS-IR, whereas in PCOS-NIR group only metformin demonstrated a significant increase (*P* < 0.01) in estradiol levels. Further metformin treated PCOS NIR cells could demonstrate significant rise (*P* < 0.01) in the levels of progesterone secretion relative to PCOS-NIR group. Testosterone levels were not detected in the cell culture supernatant. Swertiamarin could significantly down regulate expression of FSH-R and LH-R in PCOS-IR hLGCs and its effect was equally potent to metformin (Fig. 5, C and D).

## Discussion

Ever since the role of IR in the pathogenesis of PCOS has been established, a positive effect of insulin-sensitizing drugs in the treatment of PCOS has been demonstrated. In accordance with this, our study is the first one to report for the potential of swertiamarin, a bio active herbal insulin sensitizers for amelioration of IR and reestablishment of steroidogenesis in hLGC’s isolated from follicular fluid of PCOS-IR and PCOS-NIR patients using metformin as a positive control.

Insulin resistance in PCOS has been associated with increase in granulosa cell death [36]. The recovery of hLGC viability after incubation with swertiamarin in our study may be interpreted as indicative of reduced susceptibility of the PCOS-IR cells to undergo apoptosis as is observed with metformin in earlier studies [34]. However, swertiamarin did not show any effect on cell viability in hLGC from PCOS-NIR indicating the process of granulosa cell death in PCOS-NIR to be other than IR.

It is well known that ISD’s can modulate insulin signalling that can recruit its downstream docking proteins to activate several other signalling pathways in different cell types [18, 37]. Swertiamarin is well known for its anti-diabetic and anti-hyperlipidemic effects in various animal models, different cell lines and clinical trials with humans. Our results for the first time show a direct interaction of swertiamarin with key components of the classical insulin-signaling pathway thereby highlighting their ability to sensitize IR condition in hLGC’s from PCOS-IR. Moreover swertiamarin with a dose of 66 μM seemed to be a potent insulin sensitizing drug for reversing insulin sensitivity in PCOS-IR as compared to metformin 1 mM. Surprisingly metformin increased the basal expression of key signalling proteins in the insulin signalling pathway which might result in increased insulin sensitivity and decreased insulin levels to less than normal even in PCOS-NIR group in the long term.

The importance of P38 MAPK and ERK1/2 in PCOS granulosa cells has been demonstrated in studies indicating their over expression as a result of oxidative stress and inflammation leading to decrease in the expression of StAR and progesterone synthesis. [38–41]. Decrease in the protein expression of pP38 and ERK1/2 MAPK in treated hLGC’s from PCOS-IR as well as PCOS-NIR indicating reversal of oxidative stress and thus reversal of cell death. Our findings are in line with the literature where swertiamarin could enhance anti-oxidant defense system by suppressing oxidative stress and attenuate inflammation and apoptosis [30, 42].

Lipogenic genes being the regulators of the accumulation of lipids instead of cholesterol in granulosa cells, their profile was assessed. As insulin signalling is related to lipid metabolism and lipids are important for oocyte maintenance, we further observed the effect of swertiamarin on the lipogenic genes SREBP1c along with enzymes ACC-1, FAS and CPT-1 in PCOS-IR and PCOS-NIR condition. Our data indicated that SREBP-1c protein expression, in addition to the expression of lipogenic target genes ACC1 and FAS and fatty acid oxidation gene CPT-1 were suppressed by swertiamarin in hLGC’s from PCOS-IR supported by [30, 43]. The reversal of these processes explain the association of swertiamarin supplementations in decreasing lipid accumulation in granulosa cells seemed with an improvement in granulosa cell metabolism via the regulation of SREBP-1c, ACC-1, FAS and CPT-1 expression.

We further wanted to study the effect of the bio active on IGF (IGF-I, IGF-II, IGF-1R and IGF-2R) system whose involvement is appreciated in the development of preantral to preovulatory follicles, in the process of follicular atresia and is over expressed in diabetic condition [44–47]. In the present chapter, treatment of swertiamarin reversed the aberrant effects of PCOS on IGF-I, IGF-II, IGF-1R and IGF-2R in hLGC’s from PCOS-IR. Studies in literature with administration of metformin in diabetic patients have reported upregulation of IGF-1 gene and down regulation of its receptor thereby improving insulin sensitivity and glucose uptake [48–52].

As observed with the proteins involved in insulin signalling system, metformin reversed the expression of IGF-2R in hLGC’s from PCOS-NIR. These findings combined with no change in basal expression of these proteins and genes in control hLGC’s as well as PCOS-NIR hLGC’s strongly suggest that other than metformin, swertiamarin might function only during IR condition.

The process of steroidogenesis is very crucial for the development of oocyte, its fertilization and embryo implantation [53]. In PCOS condition irrespective of IR, gene and protein expression of steroidogenic factors and their corresponding steroid hormones are lowered [8, 53]. In the present study swertiamarin could revert back mRNA expression of gonadotropin receptors, mRNA and protein expression of StAR, CYP11A1, CYP19A1, 17β-HSD and 3β-HSD, their enzyme activity along with secretion of the corresponding steroid hormones estradiol and progesterone in hLGC’s from PCOS-IR thus improving the process of steroidogenesis. Metformin, the positive control of the study improves steroidogenesis by down regulating FSH-R and increasing the progesterone secretion by hLGC from PCOS women [8, 21]. Swertiamarin at a concentration of 66 μM proved to be a better drug in reversing steroidogenesis with the same potency as compared to metformin 1 mM in PCOS-IR. In the present study testosterone could not be detected in the cell culture supernatant. The finding was consistent with the literature explaining presence of the enzyme P450c17/CYp17, responsible for converting C21 steroids (progestrogens) to C19 steroids (androgens) solely in theca cells and not in granulosa cells [54].

An additional aim of this study was to determine whether swertiamarin and metformin could ameliorate decreased steroidogenesis in hLGC’s from PCOS-NIR. Strikingly, swertiamarin did not ameliorate steroidogenesis. On the basis of the findings that swertiamarin restored insulin sensitivity in PCOS-IR with no effect on PCOS-NIR, it is quite possible to speculate that swertiamarin could be mediating their effects on granulosa cell steroidogenesis only through insulin signaling. Metformin reversed back the decrease in steroidogenesis although with a less pronounced effect as compared to PCOS-IR. This effect could be attributed to the direct effect of metformin on steroidogenesis probably through a multipathway reaction with ERK1/2, pP38 MAPK or PI(3)K all involved in regulation of steroidogenesis [55–57]. Such direct effects of metformin have been supported by some clinical studies in which the insulin sensitizer increased the ovulation rate, and fertilization rates by having no effect on the basal insulin levels [13, 31, 58]. These findings indicate the possibility of some other factors in hLGC’s from PCOS-NIR yet unidentified and not associated with insulin signalling to have a role in restoring steroidogenesis by metformin. However few studies with metformin in clinical trials have reported increased ovulation rates with decreased basal insulin levels in normo insulinemic subjects indicating hypoglycaemia if prescribed to PCOS-NIR [31, 59]. Moreover as the actual treatment time for metformin to induce a clinical ovulation is 6 months, other drugs are preferred over metformin for rapidly inducing ovulation in PCOS [60]. D-chiro Inositol, an insulin sensitizer, has been observed to induce ovulation in non insulin resistant PCOS via modulating aromatase expression [61].

**Fig. 6:**
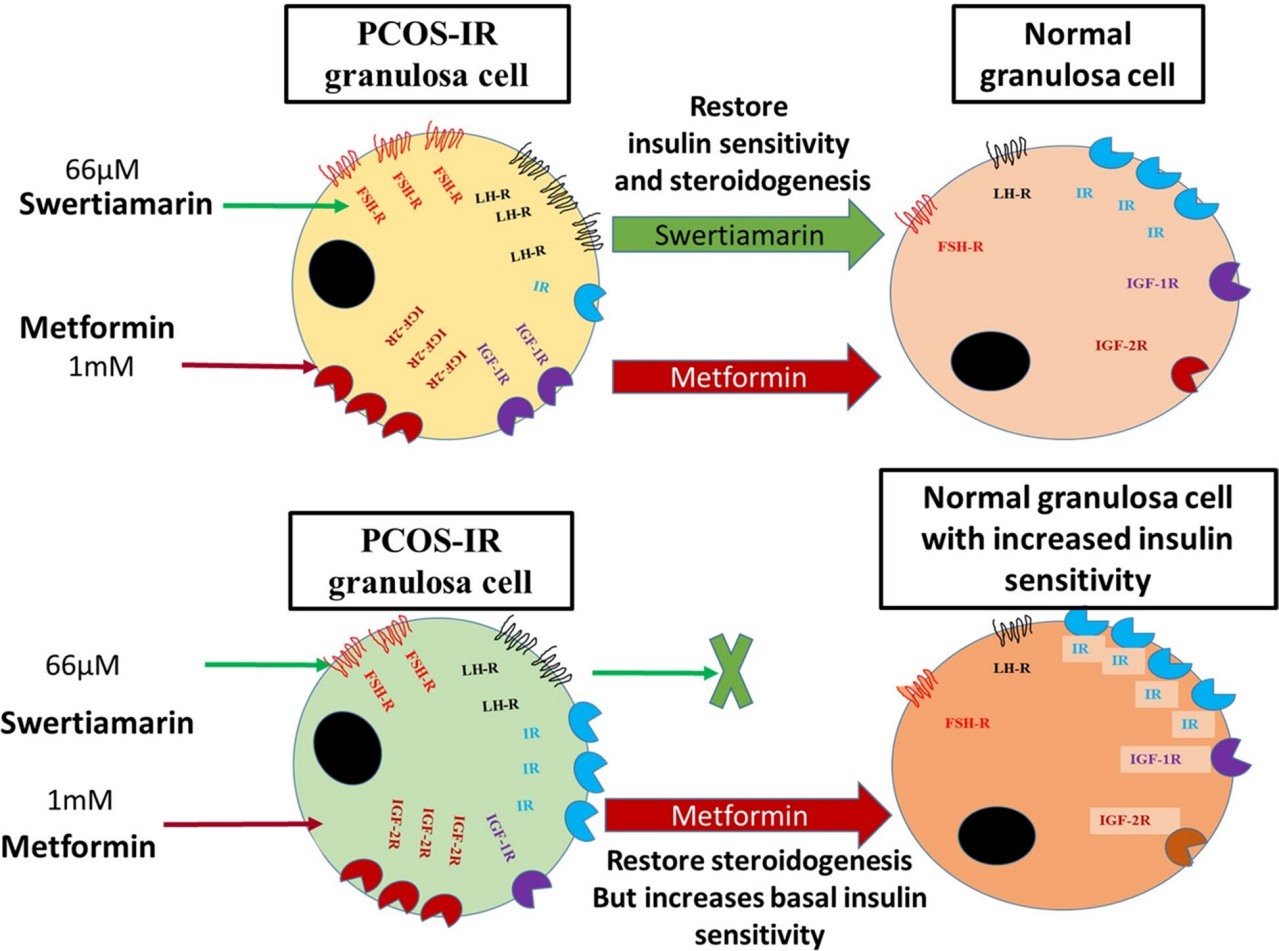
Schematic representation of the summary

Collectively the results support the notion that, swertiamarin at 66 μM show effect as insulin sensitizers for alleviating IR condition. Swertiamarin had no effect on steroidogenesis in PCOS-NIR. Metformin did restore steroidogenesis in PCOS-NIR but it is important however to highlight that it decreases the basal insulin signalling parameters in PCOS-NIR group which might lead to adverse effects in the long-term health (Fig. 6). This calls for a proper diagnosis of IR condition in PCOS so that targeted therapy can be prescribed to achieve increased pregnancy rates with decreased time.

## Supplementary Information

**Additional file 1.**

## Data Availability

All data generated or analysed during this study are included in this published article.
