# Supplementary material for: Investigating the potential role of swertiamarin on insulin resistant and non-insulin resistant granulosa cells of poly cystic ovarian syndrome patients
Source: J Ovarian Res. 2023 Mar 18;16:55. doi: 10.1186/s13048-023-01126-0 (PMC10024427; doi:10.1186/s13048-023-01126-0)
Supplement: Supplementary file 1 — Additional file 1. [file 13048_2023_1126_MOESM1_ESM.docx]

| S.No | Name | ID | Cat.No | amplicon length |
| --- | --- | --- | --- | --- |
| 1 | StAR - Steroidogenic Acute Regulatory protein | Hs00986558_g1 | 4448892 | 68 |
| 2 | CYP11A1-Cytochrome P450 side chain cleavage | Hs00897322_g1 | 4448892 | 90 |
| 3 | CYP19A1-Aromatase | Hs00903410_m1 | 4448892 | 88 |
| 4 | HSD3B2 ( 3-beta hydroxy steroid dehydrogenase type-2) | Hs01080264_g1 | 4448892 | 77 |
| 5 | HSD17B1 (17-beta hydroxy steroid dehydrogenase type-1) | Hs00907289_g1 | 4448892 | 93 |

Table 1: Taqman Gene expression probes (Human)

| **Gene** | **Accession number** | **Sequence (5'→3')** | **Product size** | **Annealing Temperature** |
| --- | --- | --- | --- | --- |
| SREBP1c | NM_001005291 | **F:** TGCATTTTCTGACACGCTTC  **R:** CCAAGCTGTACAGGCTCTCC | 171 | 60 |
| ACC1 | NM_198834 | **F:** TTTAAGGGGTGAAGAGGGTGC  **R:** CCAGAAAGACCTAGCCCTCAAG | 171 | 60 |
| FAS | NM_004104 | **F:** CACAGGGACAACCTGGAGTT  **R:** ACTCCACAGGTGGGAACAAG | 97 | 60 |
| CPT 1 | NM_001876 | **F:** TCGTCACCTCTTCTGCCTTT  **R:** ACACACCATAGCCGTCATCA | 206 | 60 |
| B-Actin | NM_001101 | **F:** ACTCTTCCAGCCTTCCTTCC  **R:** CGTACAGGTCTTTGCGGATG | 101 | 60 |

Table 2: List of primers sequences (Human) for fat metabolism with its amplicon size

| **Gene** | **Accession number** | **Sequence (5'→3')** | **Product size** | **Annealing Temperature** |
| --- | --- | --- | --- | --- |
| IGF1 | NM_000618.3 | F: AGCAGTCTTCCAACCCAATTATTTAG  R: AGATGCGAGGAGGACATGGT | 83 | 56 |
| IGF1R | NM_000875.4 | F: AAGGCTGTGACCCTCACCAT  R: CGATGCTGAAAGAACGTCCAA | 118 | 56 |
| IGF2 | NM_000612.5 | F: AGCAGTCTTCCAACCCAATTATTTAG  R: GGACTGCTTCCAGGTGTCATATT | 189 | 57 |
| IGF2R | NM_000876.2 | F: AGCAGTCTTCCAACCCAATTATTTAG  R: GAGACAAGTCAACAATAGAGCTTCCA | 197 | 60 |
| FSHR | NM_000145.3 | F: TTTCAAGAACAAGGATCCATTCC  R: CCTGGCCCTCAGCTTCTTAA | 336 | 60 |
| LHR | NM_000233.3 | F: TTCAATGGGACGACACTGACTT  R: TGTGCATCTTCTCCAGATGTACGT | 234 | 60 |

Table 3: List of primers sequences (Human) with its amplicon size

| **Name of Antibody** | **Company and Catalog No.** | **Mono/Poly clonal** | **Mol. Weight (kDa)** | **Isotype** |
| --- | --- | --- | --- | --- |
| PI(3)K  p85(19H8) | Cell Signaling #4257 | Mono | 85 | Rabbit |
| INSRB | Cell Signaling #3025 | Mono | 95 | Rabbit |
| pIRS1 | Cell Signaling #2381 | Poly | 180 | Rabbit |
| pAkt | Cell Signaling #4060 | Mono | 60 | Rabbit |
| P38 MAPK | Cell Signaling #9212 | Poly | 43 | Rabbit |
| P_44/42_ MAPK (Erk1/2) | Cell Signaling #9102 | Poly | 42,44 | Rabbit |
| Protein Kinase C-Z | Millipore  #07-264 | Poly | 72 | Rabbit |
| PPARG | Cell Signaling #2435 | Mono | 53,57 | Rabbit |
| StAR (Steroidogenic acute regulatory protein) | gifted by Prof. Stocco | Poly | 30 | Rabbit |
| CYP11A1 | Santacruz |  | 60 | Goat |
| 3BHSD | Gifted by Prof. Van Luu THE | Poly | 35 | Rabbit |
| CYP19A1 | Cell Signaling | Poly | 58 | Rabbit |
| 17BHSD | Gifted by Prof. Van Luu THE | Poly | 35 | Rabbit |
| B Actin | Thermo Scientific  #MAI-91399 | Mono | 43 | Mouse |

Table 4: Antibody for Western Blotting
